# Selection and Validation of Reference Genes for Quantitative Real-Time PCR in White Clover (*Trifolium repens* L.) Involved in Five Abiotic Stresses

**DOI:** 10.3390/plants9080996

**Published:** 2020-08-05

**Authors:** Qi Pu, Zhou Li, Gang Nie, Jiqiong Zhou, Lin Liu, Yan Peng

**Affiliations:** Department of Grassland Science, College of Animal Science and Technology, Sichuan Agricultural University, Chengdu 611130, China; puchess@163.com(Q.P.); lizhou1986814@163.com(Z.L.); nieg17@sicau.edu.cn(G.N.); jiqiong_zhou@outlook.com(J.Z.); liulinsky@126.com(L.L.)

**Keywords:** abiotic stress, gene expression, qRT-PCR, reference genes, white clover

## Abstract

White clover (*Trifolium repens* L.) is a widely cultivated cool-season perennial forage legume in temperate grassland systems. Many studies have analyzed the gene expression in this grass species using quantitative real-time reverse transcription PCR (qRT-PCR). The selection of stable reference genes for qRT-PCR is crucial. However, there was no detailed study on reference genes in different tissues of white clover under various abiotic stress conditions. Herein, 14 candidate reference genes (*ACT7, ACT101, TUA1109, TUB, CYP, 60SrRNA, UBQ, E3, GAPDH1, GAPDH2, PP2A, BAM3, SAMDC,* and *ABC*) were selected and analyzed by four programs (GeNorm, NormFinder, BestKeeper, and RefFinder). Samples were taken from two tissues (leaves and roots) under five different abiotic stresses (drought, salt, heat, cold, and heavy metal stress). Our results showed that *60SrRNA* and *ACT101* were the two top-ranked genes for all samples. Under various experimental conditions, the most stable gene was different; however, *SAMDC, UBQ, 60SrRNA,* and *ACT101* were always top ranked. The most suitable reference genes should be selected according to different plant tissues and growth conditions. Validation of these reference genes by expression analysis of *Cyt-Cu/Zn SOD* and *CAT* confirmed their reliability. Our study will benefit the subsequent research of gene function in this species.

## 1. Introduction

Quantitative real-time reverse transcription PCR (qRT-PCR) using cDNA as a template is a sensitive and powerful technique for measuring gene expression level [1]. Quantitative real-time PCR can be used not only to analyze the regulation of gene expression, monitor the expression pattern of mRNA, and quantitatively analyze the transcription level of genes, but also to conduct spatial–temporal analysis of target genes in different tissues under various treatments; as such, qRT-PCR has been widely applied in the field of molecular biology [2,3,4]. However, the quality of gene expression is affected by many experimental factors [5], and the relative quantitative method selected by researchers first needs to correct the expression amount of the target gene in the experimental process [6]. So far, using one or more reference genes is the preferred method of normalization [7], and this is also the simplest method of data processing. Accurate determination of target gene expression levels depends on the selection of stable reference genes [8,9,10].

White clover (*Trifolium repens* L.) is an important cool-season perennial legume forage which is widely cultivated in temperate grassland systems [11]. It has a high feed value and strong N fixation capacity in soil [12]. However, white clover is susceptible to drought, salt, and heat stress [13]. The understanding of expression patterns of some key genes, especially which associated with abiotic stress responses, will help in exploring the molecular mechanisms of stress response and improving the stress resistance of white clover.

Recent studies have found some suitable reference genes for plant organs and experimental conditions in different plant species [14]. To date, there has been a report on reference genes in white clover, but this report only analyzed seven candidate reference genes in two organs (leaves and stolons) under two treatments (water-limited and well-watered) [15]. Nevertheless, there is no detailed study on the reference genes in different tissues of white clover under various abiotic stresses. The ideal reference gene should be expressed stably in all cells and physiological states [16]. Genes such as *ACT* [17], *GAPDH* [18], *TUA* [19], *TUB* [20], and *EF1α* [21] are normally used as reference genes. However, some of these reference genes may have different expression among plant tissues, species, and growth conditions [22]. As no single reference gene is universally suitable for all experiments, the selection of reference genes under various experimental conditions is crucial for exactly quantifying the expression levels of genes induced by all kinds of abiotic stress [23].

In this study, we analyzed the expression stability of 14 candidate reference genes (*ACT7, ACT101, TUA1109, TUB, CYP, 60SrRNA, UBQ, E3, GAPDH1, GAPDH2, PP2A, BAM3, SAMDC,* and *ABC*) in white clover leaves and roots under drought, salt, heat, cold, and heavy metal treatments. We used four different software programs, namely GeNorm [24], NormFinder [25], BestKeeper [26] and RefFinder [27], to identify the most stable gene for qPCR. Finally, two target genes, namely *Cyt-Cu/Zn SOD* and *CAT*, from white clover were used to validate these candidate reference genes.

## 2. Materials and Methods

### 2.1. Plant Materials, Growth Conditions, and Abiotic Stress Treatments

Seeds of white clover cv. Haifa were grown in 20 × 15 × 5 cm pots containing 1 kg silica sand. Each pot was sprinkled with 0.4 g of seeds, supplemented with Hoagland’s nutrient solution, and maintained in an environmental growth chamber set to a light intensity of 100 µmol/(m·s) at 23/19 °C (day/night) with a 12-h photoperiod. One-month-old plants were used for all stress experiments. We observed the extent of five different abiotic stresses on plants, characterizing stress as mild, moderate, and severe, and then took samples. For drought stress, the plants were treated with 17% concentration of PEG6000, and samples were taken at 0, 6, 8, and 10 days. For salinity stress, the plants were treated with 250 mmol/L NaCl, and samples were taken at 0, 12, 14, and 16 days. For heat stress, plants were put in an environmental growth chamber set to 38/33 °C (day/night), and samples were taken at 0, 15, 22, and 23 days. Cold stress was imposed at 4 °C in an incubator, and samples were taken at 0, 12, 17, and 22 days. For heavy metal treatment, the nutrient solution was filled with 600 μmol/L Cd^2+^ Hoagland’s, and samples were taken at 0, 12, 17, and 22 days. Meanwhile, control plants were treated with an equal quantity of Hoagland’s nutrient solution. For all controls and treatments, the leaf and root tissues were sampled separately at three different time points with four biological replicates. All the samples were frozen immediately in liquid nitrogen and stored at −80 °C for later use.

### 2.2. RNA Isolation and cDNA Synthesis

Total RNA was extracted from the leaf and root tissues using the HiPure Universal RNA Kit (Magen Biotech Co., Ltd., China) with RNase-free DNase I (GBC, Beijing, China). RNA purity and concentration were measured with a NanoDrop ND-1000 Micro-Volume UV–Vis Spectrophotometer (NanoDrop Technologies, Wilmington, DE, USA) at 260/280 nm ratio within the range of 1.8–2.2 and 260/230 nm around 2.0. After 1% agarose gel electrophoresis, the RNA integrity was checked. Following the manufacturer’s instructions, 0.5 μg total RNA was used for first-strand cDNA synthesis using PrimeScript RT reagent Kit with gDNA Eraser (Perfect Real Time) (TaKaRa, Japan). The threefold dilution products of the generated cDNA were stored at −80 °C and used for qRT-PCR analyses.

### 2.3. Selection of Reference Genes and PCR Primer Design

Fourteen reference genes (*ACT7, ACT101, TUA1109, TUB, CYP, 60S rRNA, UBQ, E3, GAPDH1, GAPDH2, PP2A, BAM3, SAMDC,* and *ABC*) from white clover transcriptome data were selected as candidate reference genes. The target genes *Cyt-Cu/Zn SOD* and *CAT* were obtained from our research group. Primers were designed with Primer3 [28]. The primer design conditions were as follows: Tm, 59.5–62.3 °C; PCR product length, 80–199 bp; Length of primers, 20–24 bp; GC content, 45–62% (Table 1). Conventional PCR was performed to check the primers’ specificity by electrophoresis on 1.5% agarose gel.

### 2.4. qRT-PCR Analysis

qRT-PCR analyses were performed in 96-well blocks with a BIO-RAD CFX96 Real-Time PCR system (Bio-Rad, Hercules, California, USA) using NovoStart SYBR qPCR Supermix Plus (Novoprotein, China) in a 10-μL volume, containing 5 μL 2× NovoStart SYBR qPCR Supermix Plus, 1 μL diluted cDNA, 0.5 µL of forward primer (10 µmol/L), 0.5 µL of reverse primer (10 µmol/L), and 3 µL of ddH_2_O. The cycling conditions were as recommended by the manufacturer: 1 min at 95 °C, followed by 39 cycles of 95 °C for 5 s, 60 °C for 30 s, and 95 °C for 5 s. To confirm the specificity of the primers, melting curves were included after amplification. At the end of the reaction process, the melting curve was derived by heating the amplicon from 65 to 95 °C. All qRT-PCR analyses were run in technical quadruplicates and biological triplicates.

### 2.5. Stability Ranking of Candidate Reference Genes

Three different software programs (GeNorm [24], NormFinder [4], BestKeeper [26]) and the RefFinder (https://www.heartcure.com.au/reffinder/) web tool were used to evaluate the stability of 14 candidate reference genes under various stress conditions. Expression levels of the candidate reference genes were determined by quantification cycle (Cq) values. The three software programs of statistical analyses were conducted according to the manufacturer’s instructions. RefFinder was used to calculate the final rank of the 14 candidate reference genes. Results from CFX manager were exported into Microsoft Excel 2003 and transformed to create input files for each target according to the requirements of each software. For GeNorm and NormFinder, the Cq values were transformed into relative quantities using the formula 2^−ΔCq^, in which ΔCq = corresponding Cq value—minimum Cq value [29]. GeNorm identifies reference genes with good stability by calculating the M value, with a smaller M value indicating a better stability of the reference gene [26]. The program considers M values below 1.5 to indicate stable expression. The software can also calculate the V values, and the optimal number of reference genes for target gene expression normalization was decided by pairwise variation (V_n_/V_n+1_). A V_n_/V_n+1_ cutoff value of 0.15 indicates that an additional reference gene is not necessary [24,30]. The stability value calculated by NormFinder determines inter- and intragroup variation, and the lowest value indicates the highest stability. BestKeeper analyzes the stability of the candidate reference genes based on untransformed Cq values. The reference genes are considered to be the most stable when they exhibit the lowest CV and standard deviation (CV ± SD). Finally, RefFinder assigns an appropriate weight to each gene and calculates the geometric mean of their weights for the overall final ranking based on the rankings from each program.

### 2.6. Validation of Reference Genes by Expression Analysis of Cyt-Cu/Zn SOD and CAT Under Abiotic Stresses

To validate the reliability of the reference genes from software programs analysis, two target genes, namely *Cyt-Cu/Zn SOD* and *CAT*, were selected to analyze the expression patterns using the two most stable reference genes and the least stable reference gene. The results were calculated using the 2^−ΔΔCq^ method [31]. Three technical replicates were performed for each sample. The expression level of *Cyt-Cu/Zn SOD* in white clover was determined with forward primer 5′-AACTGTGTACCACGAGGACTTC-3′ and reverse primer 5′-AGACTAACAGGTGCTAACAACG-3′, while the expression level of *CAT* was determined with forward primer 5′-AACAGGACGGGAATAGCACG-3′ and reverse primer 5′-ACCAGGTTCAGACACGGAGACA-3′.

## 3. Results

### 3.1. Verification of PCR Amplicons, Primer Specificity, and Gene-Specific PCR Amplification Efficiency

**Figure 1:**
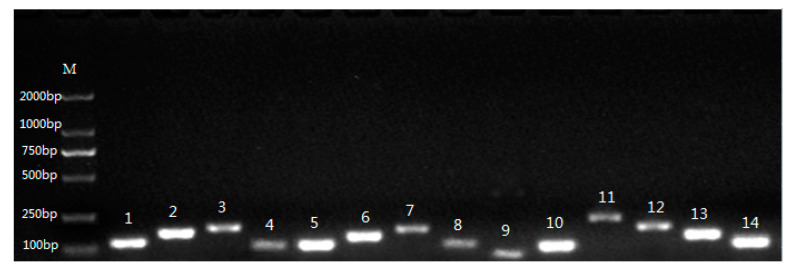
PCR products of 14 reference genes. M, DNA marker; 1, ACT7; 2, ACT101; 3, TUA; 4, TUB; 5, CYP; 6, 60S rRNA; 7, UBQ; 8, E3; 9, GAPDH1; 10, GAPDH2; 11, PP2A; 12, BAM3; 13, SAMDC; 14, ABC.

The amplicon sizes of 14 reference genes were checked by testing each primer pair using electrophoresis on a 2% agarose gel. The PCR products showed that the 80–199 bp fragments of *ACT7, ACT101, TUA1109, TUB, CYP, 60SrRNA, UBQ, E3, GAPDH1, GAPDH2, PP2A, BAM3, SAMDC,* and *ABC* were clearly amplified, consistent with the expected fragment sizes, and no impurities or primer dimers were observed (Figure 1).

**Figure 2:**
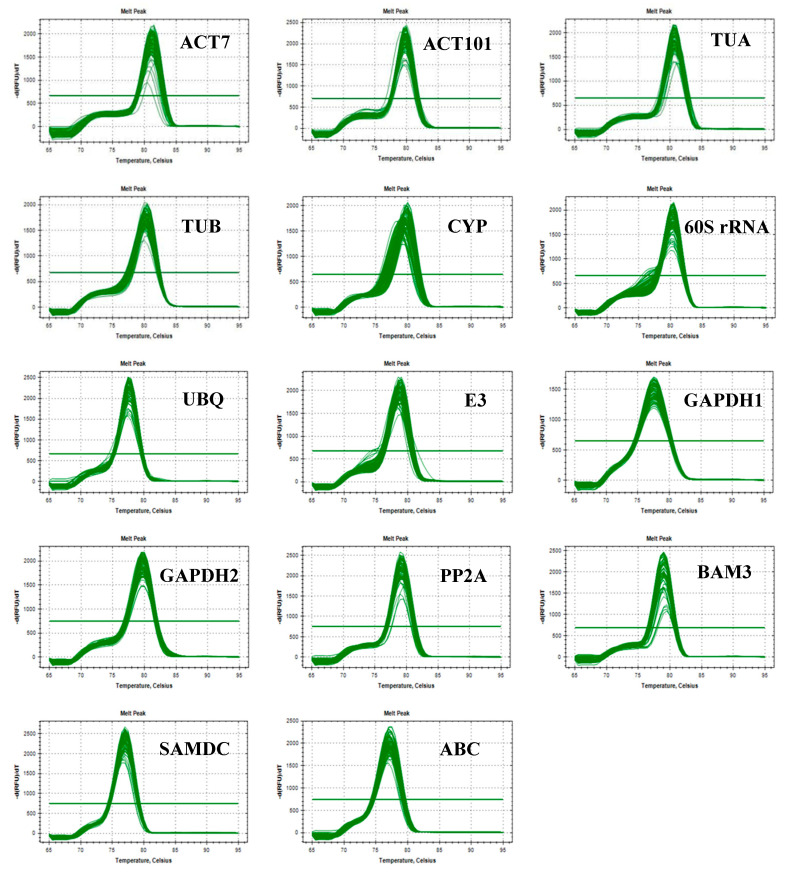
Melting curves for 14 reference genes (the horizontal lines represent baseline thresholds).

Each melting curve of 14 candidate reference genes under various abiotic stresses only exhibited a single peak, and the gene amplification curves had good repeatability (Figure 2), showing that the primers were highly specific for later qRT-PCR and the results were reliable.

**Figure 3:**
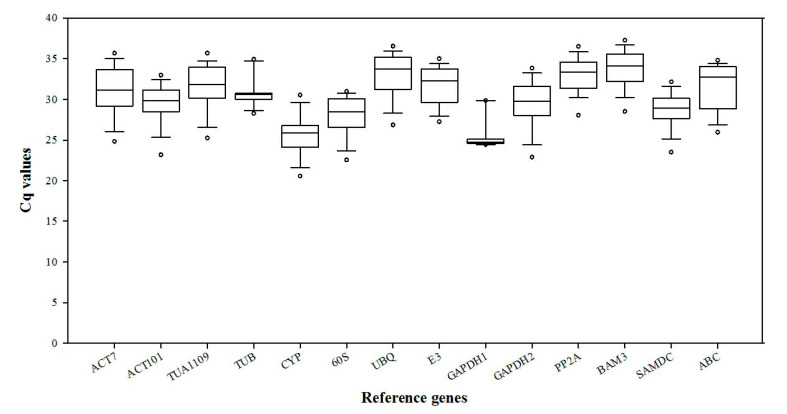
qRT-PCR Cq values for 14 candidate reference genes in white clover leaf and root samples under various abiotic stresses.

The expression levels of 14 candidate reference genes in all white clover samples are shown in the box plot (Figure 3). The quantification cycle (Cq) values ranged from 20.25 to 38.24. The lower Cq values reflect the higher mRNA transcript levels. However, under different abiotic stresses, gene expression showed different variations. Therefore, it was necessary to analyze the gene expression stability of different tissues under various abiotic stresses.

### 3.2. Stability Ranking of Candidate Reference Genes

#### 3.2.1. GeNorm Analysis

**Figure 4:**
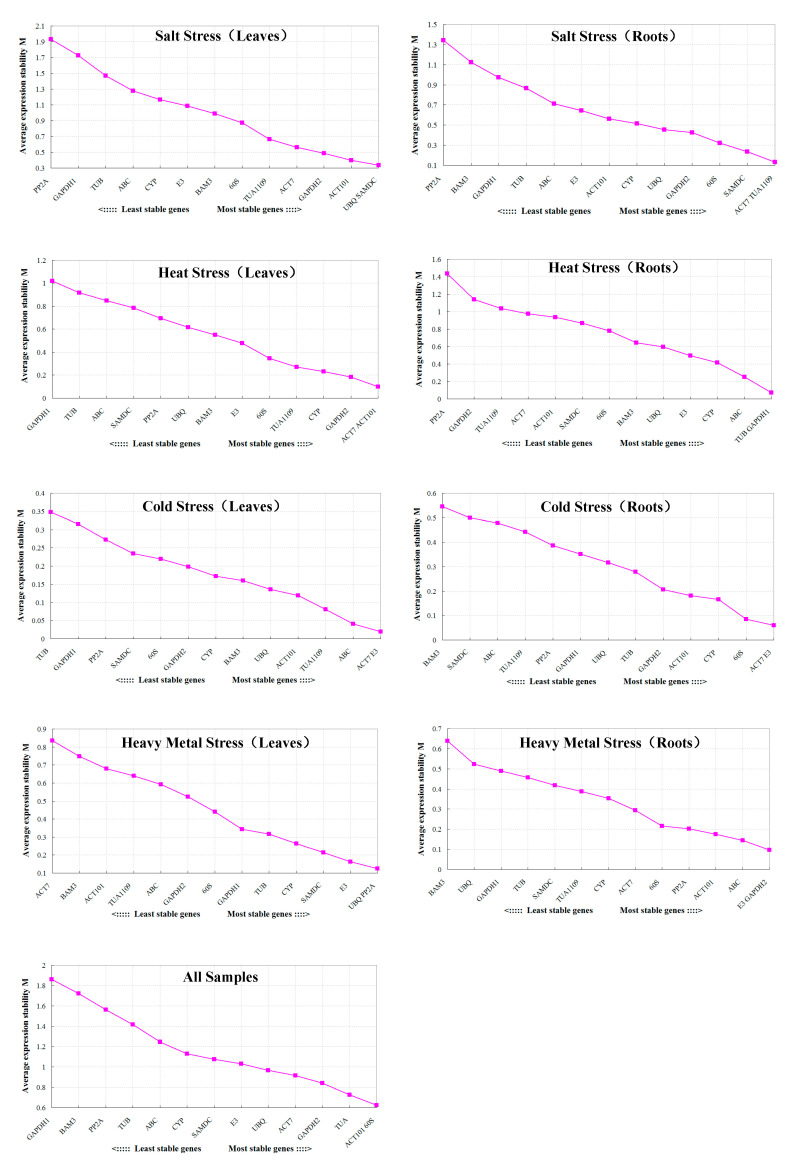
Average expression stability values (M) of candidate reference genes as determined by GeNorm analysis.

In GeNorm analysis, the M values were calculated to rank the average expression stability of 14 candidate reference genes (Figure 4). Previous studies had confirmed that an M value below the threshold of 1.5 was considered as indicative of a suitable reference gene. A lower M value represents a higher degree of expression stability for the candidate reference gene. In this study, *ACT101* and *60SrRNA* were the most stable genes for all samples, including different tissues under various abiotic stresses, while *GAPDH1* was the least stably expressed gene. For drought stress, *E3* and *SAMDC* were the most stable genes in leaf samples, while *ACT101* and *PP2A* were the most stable genes in root samples. Similarly, the *ACT101* and *SAMDC* genes ranked the highest in terms of stability for leaf samples under salt stress, while *ACT7* and *TUA1109* were the most stable for root samples. For heat stress, the *ACT7* and *ACT101* genes were the most stable in leaf samples, while the *TUB* and *GAPDH1* genes were the most stable in root samples. *ACT7* and *E3* were the most stable genes in cold-treated roots and leaves. For heavy metal stress, *UBQ* and *PP2A* were the most stable genes in leaves, while *E3* and *GAPDH2* were the most stable in roots.

**Figure 5:**
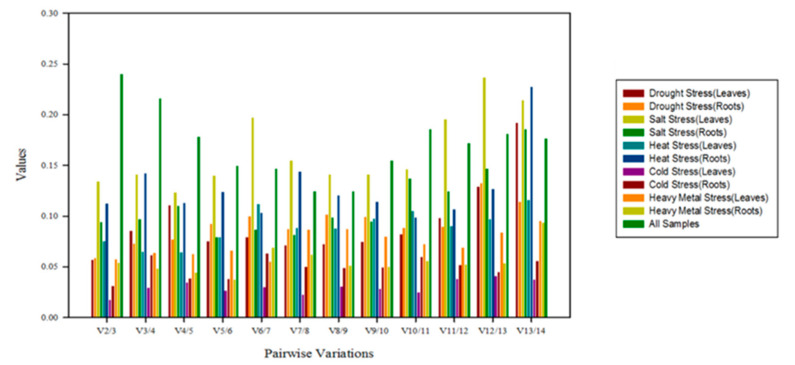
Pairwise variation (V) measure of the candidate reference genes.

GeNorm procedure was also used for determining the optimal number of reference genes required for qRT-PCR. The optimal number of reference genes for target gene expression normalization was decided by pairwise variation (V_n_/V_n+1_). A 0.15 V_n_/V_n+1_ cutoff value indicates that an additional reference gene is not necessary. In this study, except for the case of all samples, the V_2_/V_3_ values were less than 0.15 in leaves and roots under abiotic stress (Figure 5), indicating that the combination of two reference genes was suitable. However, when all samples were analyzed together to determine the optimal number of reference genes, the pairwise variation of V_2_/V_3_ was higher than 0.15, and the V_5_/V_6_ was just 0.15, indicating that five reference genes should be used for gene expression studies in white clover including various stress conditions. Thus, it was more convenient to select optimal reference genes according to different experimental conditions.

#### 3.2.2. NormFinder Analysis

The stability value calculated by NormFinder determines inter- and intragroup variation, and the lowest value means the most stable. The expression stability calculated by NormFinder for each gene showed that *E3* was the top ranked gene in leaves under drought stress, while *UBQ* was the most stable reference gene in roots under drought, heat, and cold stress and for all samples. The *60S* gene ranked highest in leaves under salt and heat stress. Meanwhile, *SAMDC* was the best in roots under salt stress, and *TUA1109* was the top ranked gene in leaves under cold stress. For heavy metal stress, *UBQ* and *ACT101* were the most stable reference genes in leaves and roots, respectively (Table 2).

#### 3.2.3. BestKeeper Analysis

BestKeeper software was used to synchronously analyze the untransformed Cq values, which reflect the stability of the candidate reference genes. The reference genes are considered to be the most stable when they exhibit the lowest CV ± SD. The results indicated that *TUB* and *GAPDH1* were the two most stable genes in our study. *GAPDH1* ranked the highest in leaves under drought, salt, heat, and cold stress and in roots under drought and salt stress. Meanwhile, *TUB* ranked the highest in leaves under heavy metal stress and roots under heat, cold, and heavy metal stress. For all samples, *TUB* was also the most stable reference gene (Table 3).

#### 3.2.4. RefFinder Analysis

Finally, RefFinder was used to assign an appropriate weight to each gene and calculate the geometric mean of their weights for the overall final ranking, based on the rankings from each program (Table 4). For all samples and drought-treated leaves, the two top-ranked genes determined by RefFinder method were the same as those determined by GeNorm. Both *UBQ* and *60S* were the most suitable reference genes in drought-treated samples. *UBQ* and *SAMDC* were identified as the most stable reference genes in leaf samples under salt stress, and *SAMDC* and *60S* were identified as the most stable reference genes in root samples under salt stress. For heat stress, the most stable combinations were *60S* plus *ACT7* in leaves and *UBQ* and *BAM3* in roots. *TUA1109* and *ACT7* were the most stable genes in cold-treated leaf samples, while *TUB* and *UBQ* were the most stable genes in cold-treated root samples. For heavy metal stress, the most stable combinations were *UBQ* plus *CYP* in leaves and *ACT101* plus *E3* in roots.

### 3.3. Validation of the Reference Genes Identified from this Study

The relative expression levels of Cyt-Cu/Zn superoxide dismutase (*SOD*) and catalase (*CAT*) genes were used to validate the performance of the identified reference genes in this study. *SOD*, which catalyzes superoxide to H_2_O_2_ and O_2_, initiates the defense system by removing superoxide, and it can be classified into three distinct groups by their metal cofactors: Cu/Zn, Mn, and Fe [32]. *Cu/Zn SOD* is present in the cytosol and chloroplasts [33]. Transgenic tobacco and cotton overexpressing chloroplastic *Cu/Zn SOD* and chloroplast-targeted *MnSOD* showed enhanced photosynthetic rates under chilling stress [34,35]. *CAT* reacts with H_2_O_2_ directly to form H_2_O and O_2_. In most species, *SOD* and *CAT* activities are relatively sensitive in response to various abiotic stresses [36].

**Figure 6:**
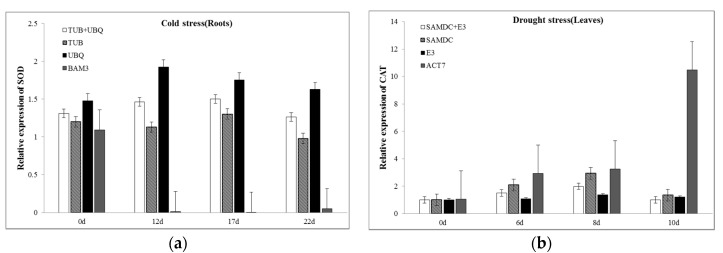
Relative expression levels of target genes. (**a**) Relative expression levels of *Cyt-Cu/Zn SOD* under cold stress using the two most stable reference genes and the least stable reference gene for normalization in white clover root tissues at different times; (**b**) relative expression levels of *CAT* under drought stress using the two most stable reference genes and the least stable reference gene for normalization in white clover leaf tissues at different times.

The relative expression levels of *Cyt-Cu/Zn SOD* and *CAT* genes were normalized using the two most stable reference genes and the least stable reference gene in white clover at different times. As shown in Figure 6, the normalized expression level of *SOD* in roots increased at 12 days and then decreased under cold treatment when using the two most stable genes (*TUB* and *UBQ*), while the expression level at 12 days was extremely low when *BAM3* was used as a reference gene. In response to drought stress, the expression levels of *CAT* in leaves were similar at 6 and 8 days. However, the relative expression decreased at 10 days when the two most stable genes (*SAMDC* and *E3*) were adopted. Meanwhile, a 10- to 11-fold higher expression level occurred at 10 days when using *ACT7* as a reference gene.

## 4. Discussion

In the process of plant growth and development, it is inevitable to face a lot of adversities. Conventional breeding techniques usually take a long time for selecting valuable genes with stable expression, but transgenic technology could improve the efficiency greatly. When studying the molecular mechanisms of stress resistance in plants and cloning stress resistance genes, real-time quantitative PCR (qRT-PCR) is needed to analyze the expression of a target gene. The accurate determination of relative gene expression mainly depends on the reference genes [37]. Therefore, the selection of suitable reference genes can reduce the experimental error [38]. Previous studies have demonstrated that there is no “universal” reference gene applicable for various experimental conditions [39]. Thus, it is necessary to select matched reference genes for quantitative real-time PCR in white clover involved in various abiotic stresses.

In this study, we observed the growth process of plants under five abiotic stresses. Leaf and root tissues under mild, moderate, and severe stress were sampled. Fourteen frequently used reference genes were picked out, and their stability was analyzed by four software programs (GeNorm, NormFinder, BestKeeperand RefFinder) under five experimental conditions (drought, salt, cold, heat, and heavy metal stress). Notably, the different algorithms evaluating the expression stability of reference genes selected different stable genes due to their different mathematical calculations [40]. Furthermore, RefFinder was used to integrate and generate the comprehensive ranking of the candidate reference genes based on the geometric mean of the weights of every gene calculated by each program [27]. The RefFinder results show us the overall ranking order, which has been widely used to select suitable reference genes in previous studies. The GeNorm results showed that it was better to select two reference genes in most experimental conditions. Finally, we concluded that the top two reference genes as ranked by the RefFinder program should be selected. In order to validate these selected candidate reference genes in white clover, the relative expression levels of *Cyt-Cu/Zn SOD* and *CAT* genes were normalized using the two most stable reference genes and the least stable reference gene. The validation results suggested that using inappropriate reference genes may significantly increase the error of target gene expression and make the results unreliable.

Furthermore, we determined that there was no single reference gene that exhibits a constant expression level in all samples of various tissues and under different experimental conditions; this was consistent with previous research [41]. Rafael Narancio [15] determined that *EF1a*, followed by *ACT11* and *UBQ*, was the most stably expressed gene across organs and treatments in white clover. From our results, *ACT* and *UBQ* also showed a high stability across most experimental conditions. However, under certain conditions, the most stable genes may be different from other species. Therefore, it is necessary to choose the most suitable reference gene for a more accurate result according to different experimental conditions, as the expression of a target gene is bound to change when using different reference genes.

In conclusion, 14 candidate reference genes were first selected in white clover. However, the optimal reference genes for different tissues (leaves and roots) under different experimental conditions are not identical. For all samples, *60SrRNA* and *ACT101* were the two top-ranked genes. Under drought stress, *SAMDC* and *UBQ* were identified as the most stable reference genes in the leaf and root samples, respectively. *UBQ, SAMDC,* and *60SrRNA* were suggested as suitable reference genes in salt stress. For heat stress, the most stable gene was *60SrRNA* in leaves, while *UBQ* was the most stable in roots. *TUA* was the most stable gene for cold-treated leaf samples, while *TUB* was the most stable gene for cold-treated root samples. For heavy metal stress, *UBQ* was the most stable gene in leaves, while *ACT101* was the most stable gene in roots. For the first time, we analyzed the most stable reference genes for different tissues in white clover under five different abiotic stresses, providing the most suitable reference for gene expression analysis in later research. This is of great significance and will be helpful in exploring the potential molecular mechanisms of the abiotic stress response in white clover.

## Figures and Tables

**Table 1:** Primer sequences for 14 reference genes used in the real-time qRT-PCR analysis.

Gene Abbreviation	Gene Name	Primer Sequence Forward and Reverse	Amplicon Length (bp)	Tm (°C)	Accession Number
ACT7	Actin 7	GGCAGACGCTGAGGATATTCAACC ATGACGTGGTCGGCCAACAATAC	124	60.3	MT822509
ACT101	Actin 101	TGCTTGATTCCGGTGATGGTGTG TTCTCGGCAGAGGTACTGAAGGAG	163	60.3	MT822510
TUA	Alpha tubulin	TGGAGGAACTGGATCTGGTCTTGG AACAGGACAGCAACATCGGTGTG	186	60.6	MT822511
TUB	Beta tubulin	CCAGCAGTACCGCAACTTGTCTG ACGACCGTGGCGTGGATCTG	94	62.3	MT822512
CYP	Cyclophilin	ACGTTGTGTTCGGTCAAGTTGTTG GGCGACGACAACAGGCTTAGAG	101	59.6	MT822513
60S rRNA	60S ribosomal RNA	AACGGTGCTGTGGAGACAATGTAC TTGTGGAACTGCTTAGTGCTCTCC	134	59.5	MT822514
UBQ	Ubiquitin	ACTGCGTGCAACCAAGGATGATAG TGCCTCGTCTGAAGACTGACCAG	163	60.0	MT822515
E3	Ubiquitin	ATTGCCTGCTGATCCTGATCTGC ACCACTGCAACCACACCAAGC	95	60.7	MT822516
GAPDH1	Glyceraldehyde 3-phosphate dehydrogenase 1	GCGTGAACGAGGCTGACTACAAG CCTTGACGATGCCGAACTTCTCC	117	60.8	MT822517
GAPDH2	Glyceraldehyde 3-phosphate dehydrogenase 2	CCATCACTGCCACTCAGAAGACTG AATGTTGAATGAGGCGGCTCTTCC	80	60.1	MT822518
PP2A	Protein phosphatase 2A	CGGAGCCGGTGTTGTGACAAG AGGCGTGCTCTGTAGGAACTCC	199	61.9	MT822519
BAM3	Beta-amylase 3	TGTTGGTGACTCATGCAGCATTCC GTGGTGTCCTTCCGGCAAGAAC	158	60.8	MT822520
SAMDC	S-adenosylmethionine decarboxylase	TCAGCAGCCAAGATGACCAACAAC ACAGCAGCACCTTCAACAGAGTTC	119	60.0	MT822521
ABC	ATP-binding	AAGGATGTACCGCGCCTTCTTATG ATCTCCGCATCTTCCGCACAATAC	82	59.5	MT822522

**Table 2:** Expression stability values for 14 white clover candidate reference genes calculated using NormFinder.

Rank	Drought Stress		Salt Stress		Heat Stress		Cold Stress		Heavy metal Stress		All Samples
	Leaves	Roots	Leaves	Roots	Leaves	Roots	Leaves	Roots	Leaves	Roots	
1	E3 (0.029)	UBQ (0.164)	60S (0.154)	SAMDC (0.136)	60S (0.106)	UBQ (0.063)	TUA1109 (0.032)	UBQ (0.061)	UBQ (0.156)	ACT101 (0.101)	UBQ (0.049)
2	SAMDC (0.029)	CYP (0.180)	UBQ (0.286)	CYP (0.136)	TUA1109 (0.156)	BAM3 (0.063)	ACT101 (0.053)	TUB (0.072)	CYP (0.177)	TUA1109 (0.124)	TUB (0.125)
3	UBQ (0.043)	60S (0.195)	CYP (0.444)	60S (0.203)	GAPDH2 (0.171)	CYP (0.136)	CYP (0.081)	PP2A (0.185)	60S (0.189)	CYP (0.140)	CYP (0.130)
4	60S (0.124)	ABC (0.299)	SAMDC (0.487)	ACT101 (0.267)	ACT7 (0.303)	ABC (0.498)	UBQ (0.100)	GAPDH1 (0.226)	PP2A (0.266)	SAMDC (0.198)	GAPDH2 (0.171)
5	CYP (0.287)	PP2A (0.431)	BAM3 (0.526)	ACT7 (0.350)	E3 (0.316)	60S (0.512)	ACT7 (0.108)	ACT101 (0.227)	E3 (0.270)	E3 (0.224)	ACT101 (0.349)
6	GAPDH2 (0.307)	TUB (0.432)	E3 (0.564)	TUA1109 (0.451)	CYP (0.326)	SAMDC (0.544)	E3 (0.111)	ACT7 (0.255)	GAPDH2 (0.372)	PP2A (0.229)	TUA (0.414)
7	ACT101 (0.477)	GAPDH2 (0.514)	ACT101 (0.686)	GAPDH2 (0.576)	ACT101 (0.384)	E3 (0.553)	ABC (0.115)	CYP (0.272)	GAPDH1 (0.388)	GAPDH2 (0.291)	GAPDH1 (0.487)
8	PP2A (0.506)	ACT101 (0.536)	GAPDH2 (0.919)	UBQ (0.644)	BAM3 (0.401)	GAPDH1 (0.595)	BAM3 (0.137)	60S (0.291)	TUB (0.405)	ACT7 (0.295)	PP2A (0.526)
9	TUA1109 (0.538)	E3 (0.565)	ACT7 (1.029)	TUB (0.657)	UBQ (0.444)	TUB (0.646)	GAPDH2 (0.164)	E3 (0.300)	SAMDC (0.418)	ABC (0.295)	SAMDC (0.527)
10	ABC (0.584)	TUA1109 (0.662)	TUB (1.257)	GAPDH1 (0.754)	PP2A (0.656)	ACT101 (0.656)	60S (0.248)	TUA1109 (0.342)	ABC (0.430)	60S (0.328)	ABC (0.607)
11	BAM3 (0.604)	SAMDC (0.700)	TUA1109 (1.279)	E3 (0.816)	TUB (0.765)	ACT7 (0.688)	PP2A (0.256)	ABC (0.349)	TUA1109 (0.464)	TUB (0.335)	E3 (0.649)
12	ACT7 (0.891)	ACT7 (0.765)	ABC (1.288)	ABC (1.025)	SAMDC (0.859)	TUA1109 (0.919)	SAMDC (0.257)	SAMDC (0.362)	ACT101 (0.547)	GAPDH1 (0.386)	ACT7 (0.708)
13	TUB (1.008)	GAPDH1 (1.103)	GAPDH1 (1.979)	BAM3 (1.182)	ABC (0.873)	GAPDH2 (1.283)	GAPDH1 (0.335)	GAPDH2 (0.399)	BAM3 (0.813)	UBQ (0.532)	60S (0.773)
14	GAPDH1 (1.871)	BAM3 (1.112)	PP2A (2.064)	PP2A (1.794)	GAPDH1 (1.117)	PP2A (2.200)	TUB (0.358)	BAM3 (0.537)	ACT7 (0.914)	BAM3 (0.906)	BAM3 (2.841)

**Table 3:** Expression stability values for 14 white clover candidate reference genes calculated using BestKeeper.

Rank	Drought Stress		Salt Stress		Heat Stress		Cold Stress		Heavy Metal Stress		All Samples
	Leaves	Roots	Leaves	Roots	Leaves	Roots	Leaves	Roots	Leaves	Roots	
1	GAPDH1 (1.25 ± 0.31)	GAPDH1 (0.73 ± 0.18)	GAPDH1 (0.25 ± 0.06)	GAPDH1 (0.01 ± 0.00)	GAPDH1 (0.20 ± 0.05)	TUB (0.20 ± 0.06)	GAPDH1 (0.03 ± 0.01)	TUB (0.19 ± 0.07)	TUB (0.02 ± 0.01)	TUB (0.06 ± 0.02)	TUB (4.73 ± 1.47)
2	TUB (2.28 ± 0.67)	BAM3 (1.33 ± 0.46)	PP2A (1.55 ± 4.81)	TUB (0.35 ± 0.11)	TUB (1.06 ± 0.32)	GAPDH1 (0.38 ± 0.09)	TUB (0.18 ± 0.06)	GAPDH1 (0.24 ± 0.07)	GAPDH1 (0.32 ± 0.08)	GAPDH1 (0.18 ± 0.04)	PP2A (5.39 ± 1.78)
3	E3 (5.89 ± 1.74)	TUB (2.27 ± 0.67)	BAM3 (2.26 ± 6.73)	BAM3 (1.40 ± 0.48)	ACT101 (2.42 ± 0.71)	ABC (0.89 ± 0.30)	PP2A (0.48 ± 0.16)	PP2A (0.48 ± 0.16)	CYP (0.76 ± 0.20)	SAMDC (0.50 ± 0.14)	BAM3 (5.44 ± 1.84)
4	BAM3 (5.91 ± 1.75)	E3 (2.50 ± 0.69)	SAMDC (2.48 ± 8.60)	ACT101 (2.82 ± 0.83)	ACT7 (2.61 ± 0.78)	E3 (1.71 ± 0.56)	UBQ (0.87 ± 0.29)	UBQ (0.55 ± 0.20)	E3 (0.78 ± 0.26)	TUA1109 (0.64 ± 0.20)	SAMDC (6.17 ± 1.77)
5	60S (6.15 ± 1.56)	ABC (2.61 ± 0.73)	ABC (2.78 ± 9.20)	SAMDC (3.20 ± 0.94)	TUA1109 (2.99 ± 0.92)	UBQ (1.99 ± 0.68)	ACT101 (0.92 ± 0.29)	ACT101 (0.90 ± 0.28)	UBQ (0.97 ± 0.35)	CYP (0.91 ± 0.23)	E3 (6.20 ± 1.97)
6	UBQ (6.20 ± 1..80)	UBQ (3.86 ± 1.08)	TUB (2.80 ± 0.84)	PP2A (3.49 ± 1.15)	GAPDH2 (3.03 ± 0.88)	CYP (2.16 ± 0.57)	TUA1109 (1.01 ± 0.34)	CYP (1.03 ± 0.31)	SAMDC (1.05 ± 0.32)	BAM3 (1.54 ± 0.52)	GAPDH1 (6.32 ± 1.63)
7	CYP (6.21 ± 1.38)	CYP (4.73 ± 1.03)	E3 (6.16 ± 1.93)	60S (3.53 ± 0.98)	CYP (3.36 ± 0.84)	BAM3 (2.23 ± 0.81)	ACT7 (1.30 ± 0.43)	ABC (1.11 ± 0.38)	PP2A (1.06 ± 0.38)	ACT7 (1.66 ± 0.51)	ACT101 (6.85 ± 2.01)
8	PP2A (6.71 ± 2.09)	PP2A (5.12 ± 1.52)	CYP (6.59 ± 1.61)	ACT7 (3.57 ± 1.11)	60S (4.13 ± 1.16)	SAMDC (3.78 ± 1.16)	E3 (1.34 ± 0.43)	ACT7 (1.16 ± 0.41)	BAM3 (1.21 ± 0.41)	PP2A (1.72 ± 0.60)	UBQ (7.14 ± 2.35)
9	SAMDC (6.91 ± 1.81)	60S (5.48 ± 1.28)	60S (6.65 ± 1.86)	CYP (3.72 ± 0.94)	BAM3 (4.26 ± 1.43)	ACT7 (4.07 ± 1.34)	GAPDH2 (1.36 ± 0.41)	BAM3 (1.25 ± 0.46)	ABC (2.04 ± 0.69)	ACT101 (1.78 ± 0.52)	60S (7.21 ± 2.02)
10	TUA1109 (7.99 ± 2.25)	TUA1109 (5.90 ± 1.58)	UBQ (6.80 ± 2.24)	TUA1109 (3.76 ± 1.19)	E3 (4.79 ± 1.48)	60S (4.37 ± 1.29)	BAM3 (1.38 ± 0.43)	E3 (1.28 ± 0.45)	60S (2.06 ± 0.63)	E3 (1.85 ± 0.61)	TUA1109 (7.35 ± 2.31)
11	ABC (8.02 ± 2.21)	GAPDH2 (6.18 ± 1.52)	ACT101 (8.65 ± 2.57)	UBQ (3.92 ± 1.32)	UBQ (4.80 ± 1.54)	ACT101 (4.55 ± 1.40)	CYP (1.40 ± 0.38)	GAPDH2 (1.29 ± 0.42)	GAPDH2 (2.66 ± 0.83)	ABC (2.03 ± 0.69)	CYP (7.48 ± 1.90)
12	GAPDH2 (8.09 ± 2.06)	ACT101 (6.40 ± 1.60)	ACT7 (8.84 ± 2.72)	GAPDH2 (4.32 ± 1.27)	PP2A (5.02 ± 1.70)	TUA1109 (4.62 ± 1.50)	ABC (1.43 ± 0.43)	SAMDC (1.31 ± 0.40)	TUA1109 (2.68 ± 0.90)	GAPDH2 (2.23 ± 0.65)	ACT7 (7.70 ± 2.40)
13	ACT101 (8.37 ± 2.19)	ACT7 (6.59 ± 1.79)	GAPDH2 (9.52 ± 2.82)	E3 (4.97 ± 1.58)	ABC (6.66 ± 2.02)	PP2A (4.74 ± 1.55)	60S (1.79 ± 0.53)	TUA1109 (1.33 ± 0.46)	ACT101 (3.15 ± 1.00)	60S (2.52 ± 0.70)	GAPDH2 (7.84 ± 2.30)
14	ACT7 (9.56 ± 2.63)	SAMDC (7.12 ± 1.79)	TUA1109 (9.72 ± 3.11)	ABC (5.23 ± 1.71)	SAMDC (6.89 ± 1.98)	GAPDH2 (6.33 ± 2.05)	SAMDC (1.93 ± 0.55)	60S (1.53 ± 0.46)	ACT7 (3.91 ± 1.31)	UBQ (2.71 ± 0.92)	ABC (8.33 ± 2.61)

**Table 4:** Expression stability values for white clover candidate reference genes calculated using RefFinder.

Treatment	Ranking order													
	1	2	3	4	5	6	7	8	9	10	11	12	13	14
All samples	60S	ACT101	E3	SAMDC	CYP	TUA1109	TUB	UBQ	GAPDH2	ACT7	GAPDH1	PP2A	BAM3	ABC
Drought stress (Leaves)	SAMDC	E3	UBQ	60S	GAPDH2	CYP	GAPDH1	ACT101	TUB	TUA1109	PP2A	BAM3	ABC	ACT7
Drought stress (Roots)	UBQ	60S	PP2A	CYP	ACT101	ABC	GAPDH2	TUB	GAPDH1	E3	BAM3	SAMDC	TUA1109	ACT7
Salt stress (Leaves)	UBQ	SAMDC	60S	ACT101	CYP	BAM3	GAPDH1	E3	TUB	GAPDH2	ACT7	PP2A	TUA1109	ABC
Salt stress (Roots)	SAMDC	60S	ACT7	TUA1109	CYP	ACT101	GAPDH1	TUB	GAPDH2	UBQ	BAM3	E3	ABC	PP2A
Heat stress (Leaves)	60S	ACT7	GAPDH2	ACT101	TUA1109	CYP	E3	GAPDH1	TUB	BAM3	UBQ	PP2A	SAMDC	ABC
Heat stress (Roots)	UBQ	BAM3	TUB	GAPDH1	CYP	ABC	E3	60S	SAMDC	ACT101	ACT7	TUA1109	GAPDH2	PP2A
Cold stress (Leaves)	TUA1109	ACT7	ACT101	E3	CYP	UBQ	ABC	GAPDH1	BAM3	PP2A	TUB	GAPDH2	60S	SAMDC
Cold stress (Roots)	TUB	UBQ	ACT7	ACT101	PP2A	GAPDH1	E3	CYP	60S	ABC	GAPDH2	SAMDC	TUA1109	BAM3
Heavy metal stress (Leaves)	UBQ	CYP	PP2A	E3	TUB	GAPDH1	60S	SAMDC	GAPDH2	ABC	TUA1109	BAM3	ACT101	ACT7
Heavy metal stress (Roots)	ACT101	E3	TUA1109	CYP	GAPDH2	SAMDC	TUB	PP2A	ABC	GAPDH1	ACT7	60S	BAM3	UBQ
